# Understanding the Role of Accredited Drug Dispensing Outlets in Tanzania’s Health System

**DOI:** 10.1371/journal.pone.0164332

**Published:** 2016-11-08

**Authors:** Martha Embrey, Catherine Vialle-Valentin, Angel Dillip, Bernard Kihiyo, Romuald Mbwasi, Innocent A. Semali, John C. Chalker, Jafary Liana, Rachel Lieber, Keith Johnson, Edmund Rutta, Suleiman Kimatta, Elizabeth Shekalaghe, Richard Valimba, Dennis Ross-Degnan

**Affiliations:** 1 Pharmaceuticals & Health Technologies Group, Management Science for Health, Arlington, Virginia, United States of America; 2 Drug Policy Research Group, Department of Population Medicine, Harvard Pilgrim Health Care Institute, Harvard Medical School, Boston, Massachusetts, United States of America; 3 Ifakara Health Institute, Dar es Salaam, Tanzania; 4 Tanzania Consumer Advocacy Society, Dar es Salaam, Tanzania; 5 Apotheker Consultancy (T) Limited, St. Johns University of Dodoma, Dodoma, Tanzania; 6 School of Public Health, Muhimbili University of Health and Allied Sciences, Dar es Salaam, Tanzania; 7 Pharmaceuticals & Health Technologies Group, Management Science for Health, Dar es Salaam, Tanzania; 8 Pharmacy Council of Tanzania, Dar es Salaam, Tanzania; Centre for Geographic Medicine Research Coast, KENYA

## Abstract

**Introduction:**

People in many low-income countries access medicines from retail drug shops. In Tanzania, a public-private partnership launched in 2003 used an accreditation approach to improve access to quality medicines and pharmaceutical services in underserved areas. The government scaled up the accredited drug dispensing outlet (ADDO) program nationally, with over 9,000 shops now accredited. This study assessed the relationships between community members and their sources of health care and medicines, particularly antimicrobials, with a specific focus on the role ADDOs play in the health care system.

**Methods:**

Using mixed methods, we collected data in four regions. We surveyed 1,185 households and audited 96 ADDOs and 84 public/nongovernmental health facilities using a list of 17 tracer drugs. To determine practices in health facilities, we interviewed 1,365 exiting patients. To assess dispensing practices, mystery shoppers visited 306 ADDOs presenting one of three scenarios (102 each) about a child’s respiratory symptoms.

**Results and Discussion:**

Of 614 household members with a recent acute illness, 73% sought outside care—30% at a public facility and 31% at an ADDO. However, people bought medicines more often at ADDOs no matter who recommended the treatment; of the 581 medicines that people had received, 49% came from an ADDO. Although health facilities and ADDOs had similar availability of antimicrobials, ADDOs had more pediatric formulations available (p<0.001). The common perception was that drugs from ADDOs are more expensive, but the difference in the median cost to treat pneumonia was relatively minimal (US$0.26 in a public facility and US$0.30 in an ADDO). Over 20% of households said they had someone with a chronic condition, with 93% taking medication, but ADDOs are allowed to sell very few chronic care-related medicines. ADDO dispensers are trained to refer complicated cases to a health facility, and notably, 99% of mystery shoppers presenting a pneumonia scenario received an antimicrobial (54%), a referral (90%), or both (45%), which are recommended practices for managing pediatric pneumonia. However, one-third of the dispensers needlessly sold antibiotics for cold symptoms, and 85% sold an antibiotic on request. In addition, the pneumonia scenario elicited more advice on handling the illness than the cold symptoms scenario (61% vs. 15%; p<0.0001), but overall, only 44% of the dispensers asked any of the shoppers about danger signs potentially associated with pneumonia in a child.

**Conclusion:**

ADDOs are the principal source of medicines in Tanzania and an important part of a multi-faceted health care system. Poor prescribing in health facilities, poor dispensing at ADDOs, and inappropriate patient demand continue to contribute to inappropriate medicines use. Therefore, while accreditation has attempted to address the quality of pharmaceutical services in private sector drug outlets, efforts to improve access to and use of medicines in Tanzania need to target ADDOs, public/nongovernmental health facilities, and the public to be effective.

## Introduction

Governments and their development partners have emphasized public sector health services in low-income countries for many years, focusing most of their attention and resources on improving performance in that sector. However, recognition of the role of private sector providers in the overall health landscape has been growing [1]. Retail drug sellers in particular are widely chosen as a source of care by many consumers in low-income settings, especially those in rural or peri-urban areas lacking easy access to full-service pharmacies [2–3]. Historically in Tanzania, the Tanzania Food and Drugs Authority and its predecessor agency authorized retail drug shops, known as Part II shops, to sell only nonprescription medicines. With more than 5,600 stores registered in 2003, and many more operating without registration, the shops constituted the largest and most popular private sector source of medicines in Tanzania—popular primarily because they were located nearby, had more convenient hours, and were better stocked than public sector clinics [4–6]. However, some of the problems with Part II shops included illegal sales of prescription-only medicines, untrained and unqualified drug sellers, and lack of regulatory monitoring and enforcement [7–8].

To address these issues, a public-private initiative that aimed to improve the quality of products and services at Part II shops was launched in 2003. The accredited drug dispensing outlet (ADDO) program took a comprehensive approach that combined owner and dispenser training, government accreditation based on standards, business incentives, and local regulatory enforcement, with efforts to increase consumer demand for quality products and services [9]. The government of Tanzania has now rolled out the ADDO program in all mainland districts with more than 9,000 shops accredited and over 19,000 dispensers trained. This compares with about 8,000 public and private health facilities of all levels of care. The accreditation program’s primary business incentive allows ADDOs to dispense select prescription-only medicines in addition to those available over-the-counter. However, owners report that the benefit they value the most is the dispenser training [10].

The commonly used prescription-only medicines that ADDOs are allowed to sell include antimicrobials such as amoxicillin, co-trimoxazole, and erythromycin; the recommended first-line antimalarial, artemether-lumefantrine; and quinine for severe malaria. On the ADDO list of approved medicines, products for chronic illnesses are limited to propranolol for hypertension and heart arrhythmia; bendrofluazide, a diuretic for hypertension; aminophylline for asthma; phenytoin for seizure disorders, such as epilepsy; and anti-inflammatory/pain medicines, such as diclofenac and indomethacin. S1 Table includes the full list of ADDO-approved medicines.

As a popular hub for community health care, ADDO dispensers represent a new class of community-level health care workers who are trained to assess health situations and either sell appropriate medicines for simple conditions or refer customers to the nearest health facility for complicated illnesses [9]. The dispensers also fill prescriptions that customers bring in from the public/nongovernmental organization (NGO) health facility. Because they function as part of the health system, ADDOs have been used as a platform for public health interventions, including increasing access to artemisinin-based combination therapy for malaria, and they have been incorporated into multiple public health strategies, from family planning to achievement of the Millennium Development Goals [11–13].

A number of studies in Tanzania have documented individual components of community medicines use, such as care-seeking behavior, dispensing practices in Part II shops or ADDOs, or public sector prescribing practices [14–15]. None has taken an all-inclusive approach to assessing the relationships between medicines and their sources in Tanzanian communities, with a specific focus on the role of ADDOs in the health care system and on access to and use of antimicrobials. Because accredited shops are allowed to sell selected antimicrobials, we were interested in practices that might lead to a higher risk of antimicrobial resistance.

The purpose of this study was to use a mixed methods cross-sectional assessment to explore the relationships among different components that contribute to antimicrobial access and use in Tanzania with a particular focus on ADDOs. The components and our data collection methodologies included consumer care-seeking and medicines use (household survey); health facility prescribing and dispensing practices (interviews with patients leaving health facilities); ADDO dispensing practices (mystery shopper exercise); and availability and prices of antimicrobials (audits of ADDOs and health facilities). To determine stakeholder knowledge and attitudes about important issues and to help characterize behaviors, practices, and perceptions, particularly as they relate to the role of ADDOs, we conducted a qualitative study that has been published elsewhere [16]. These qualitative results provide context in the discussion for the current study findings.

## Methods

### Location

We purposively selected four diverse regions for the assessment: Morogoro, located in east central Tanzania, which has had ADDOs since 2006; Singida, a relatively low-income region in the west central area with ADDOs since 2008; Mbeya, a relatively wealthy southwestern region with ADDOs for the previous three years; and Tanga, a northeastern region which had had ADDOs for two years. Within each region, we randomly selected three districts, then five wards within each district, both with probability proportional to population size, for a total of 60 wards. Within each sample ward, we randomly selected ADDOs and public/NGO health facilities from the lists of those operating at the time of the survey. We pre-tested all the data collection instruments in non-study districts; the data were then collected in all four regions in April (mystery shoppers), May (shop and health facility audits), and June (household surveys) of 2013.

### Household survey

A household survey characterized medicines access, use, and caregiving in the community. Within each sample ward, we randomly selected four villages and then randomly selected five households within each village, for a planned sample of 1,200 households. A household was defined as people eating from the same pot. The households in each village were first listed and divided into five strata; a random start in the first stratum was obtained using the last two numbers of the village chairperson’s cell phone number, and systematic sampling was used to obtain the other four households in the village.

Data collectors asked to speak with an adult at least 18 years old who met at least three of the following criteria: main health care decision maker, most knowledgeable about health of household members, most knowledgeable about health expenditures of the household, most knowledgeable about health utilization by household members, or designated care giver for sick household members. Respondents were asked whether anyone in the household had had an acute illness in the previous two weeks or had a current chronic illness defined as “an illness that will not go away or takes a long time to go away, even when treated.” If so, we assessed whether and where each person had sought advice, care, and medicines for the illness. Data collectors also examined the households’ stock of medicines and asked respondents about their views on the quality of service at ADDOs and public health facilities and about their knowledge of antimicrobials.

### Facility and ADDO audits and exit interviews

To examine availability and price of antimicrobials in ADDOs, we randomly selected 96 ADDOs from the different wards. We also randomly selected one or two public health care facilities (hospitals, health centers, dispensaries) and up to one NGO facility in each ward as available, for a total of 98 primary care facilities. At the health care facilities, 1,365 exiting patients were selected as a convenience sample during study team visits to assess prescribing and dispensing practices. Up to 30 patients presenting for care at each facility on the day of the survey were randomly interviewed upon exit from the dispensing area or facility pharmacy.

In each ADDO and public/NGO health facility, we checked drug stocks and selling prices for a tracer list of 17 medications (S1 Table); our tracer list focused on antimicrobials, because our study objectives included an interest in practices that may lead to antimicrobial resistance. The price ratio for each product was calculated as the median price charged at each outlet (ADDOs and health facilities) compared to the price list from the Medical Stores Department, which is Tanzania’s parastatal pharmaceutical supplier for the public sector.

### Mystery shoppers

To assess ADDO performance in treating respiratory infections and dispensing practices, we conducted 306 “mystery shopper” visits in 306 ADDOs [17]. Members of the Tanzania Consumer Advocacy Society from each study region were trained to pose as a parent or caregiver of a one-year-old child, who was at home. Each ADDO was visited once by a mystery shopper acting out one of three scenarios (102 visits each): 1) pneumonia (child with cough, difficulty breathing, and fast breathing with harsh noise); 2) mild acute respiratory infection (ARI) (child with cough and runny nose); and 3) mild ARI with a request for an antibiotic (Septrin^®^, a brand of co-trimoxazole that is widely used locally). The mystery shopper visits occurred at the same ADDOs that were selected for the facility audits, but took place the month before those data collection activities to avoid raising suspicion among shop staff. The mystery shopper reported to the data collector directly after a visit to avoid mixing up or forgetting information. The mystery shopper responded to questions on a standardized form that the data collector recorded on paper and later transferred to an electronic form.

### Statistical analysis

Data from the household, health facility, and ADDO surveys were analyzed with STATA 12 survey commands that use sampling weights to adjust for the complex survey sample design (StataCorp LP, College Station, Texas). The four purposively selected regions were treated with weights equal to their populations in calculating sample-wide estimates. Sampling weights (inverse of the sampling fractions) from each of the three stages of the sample were applied in the analyses of the household survey and the facility medication availability data. Data on the median, 25^th^ percentile, and 75^th^ percentile of medicine prices were derived from the unweighted data. Household and health facility survey data are presented as percentage estimates with 95% confidence intervals.

The mystery shopper visits and exit interviews did not use a probability sample and were analyzed without weights. For each type of visit or interview, we present data as percentages. Statistical comparison of results across scenarios in the mystery shopper interviews, household survey, and other select comparisons were performed with a chi-square test (non-weighted samples) or Pearson chi-square (weighted samples).

For this study, our classification of antimicrobials included metronidazole, but did not include antimalarials or antiretrovirals.

### Ethical clearance

We obtained ethical clearance for the study from Tanzania’s National Institute for Medical Research and the Harvard Pilgrim Health Care Institutional Review Board. Household survey respondents and ADDO owners and dispensers whom we interviewed were informed about the study and its objectives and signed consent forms indicating their willingness to participate.

## Results

### Household reported illnesses and care-seeking habits

We collected data from 1,185 households with 6,384 members that characterized the types of illnesses and care-seeking practices in the community. S2 Table presents the characteristics of the household members.

#### Acute illness

In the survey, 614 household members (10%) from 480 households (40%) had had an acute illness in the previous two weeks (Table 1). Cough was the leading symptom (50%) followed by symptoms of fever (47%), pain (22%), and thirst/sweating (15%). For the subgroup of children under five years with an illness in the previous two weeks, 66% had symptoms of ARI, with 32% reporting fever.

**Table 1:** Characteristics of Household Members with Recent Acute Illness by Age.

Individuals with acute illness(es)	All	<5 years	5–14 years	15+ years	p-value*
	n = 606	n = 123	n = 144	n = 339	
	Weighted % [95%CI]	Weighted % [95%CI]	Weighted % [95%CI]	Weighted % [95%CI]	
**Region**					
Mbeya	48.4 [30.2, 67.1]	50.7 [28.2, 72.9]	53.4 [32.1, 73.5]	45.1 [28.2, 63.3]	0.314
Morogoro	19.9 [12.6, 30.2]	18.2 [8.6, 34.5]	18.8 [10.2, 32.1]	21.1 [13.6, 31.3]	0.746
Singida	9.8 [5.5, 16.9]	10.1 [4.5, 21.2]	8.5 [3.9, 18.1]	10.2 [5.9, 17.0]	0.788
Tanga	21.9 [13.7, 33.0]	21.0 [10.5, 37.5]	19.2 [10.3, 32.8]	23.6 [14.8, 35.3]	0.556
**Severe illness**	9.3 [5.8, 14.5]	11.6 [6.2, 20.7]	3.5 [1.2, 9.7]	11.3 [6.3, 19.5]	0.062
**Symptoms**					
Cold-cough, runny nose	50.2 [40.1, 60.2]	65.9 [55.6, 74.9]	51.9 [33.7, 69.7]	43.6 [32.6, 55.2]	0.025
Malaria-fever, headache, hot body	46.8 [38.1, 55.7]	32.2 [14.3, 57.5]	44.5 [30.0, 60.0]	53.6 [36.0, 70.3]	0.252
Pain, aches	22.4 [16.7, 29.5]	16.9 [6.3, 37.9]	27.5 [17.5, 40.5]	22.0 [12.7, 35.5]	0.527
Thirst, sweating	15.1 [8.7, 24.8]	18.0 [8.5, 34.2]	15.0 [5.4, 35.2]	14.0 [8.5, 22.2]	0.712
Watery diarrhea	5.8 [3.4, 9.9]	11.0 [6.0, 19.2]	5.5 [1.6, 17.3]	4.0 [1.6, 9.5]	0.170
**Sought care**					
Outside the home	68.6 [56.3, 78.8]	71. [47.7, 87.3]	76.6 [65.4, 85.1]	63.6 [47.4, 77.2]	0.217
In ADDO/drug store	30.9 [24.5, 38.1]	28.2 [18.5, 40.4]	36.0 [24.4, 49.4]	29.4 [21.8, 38.4]	0.414
In public health center or dispensary	18.3 [12.2, 26.4]	23.8 [14.0, 37.5]	20.1 [10.6, 34.8]	15.2 [9.6, 23.1]	0.187
In public hospital	12.0 [8.4, 16.9]	14.6 [6.8, 28.6]	12.7 [3.8, 34.9]	10.6 [6.2, 17.6]	0.784
In private facility (profit or nonprofit)	11.1 [6.2, 19.0]	14.6 [5.8, 32.1]	12.6 [5.0, 28.5]	9.0 [5.4, 14.5]	0.361
Elsewhere**	8.0 [4.8, 13.0]	6.7 [1.9, 20.5]	13.9 [6.4, 27.6]	5.5 [3.1, 9.7]	0.091
**Visited ADDO as first source of care**	17.1 [12.5, 23.1]	13.1 [7.4, 22.1]	20.4 [12.3, 32.0]	17.1 [11.7, 24.2]	0.329
**Received referral from the ADDO**	3.7 [1.6, 8.2]	0.6 [0.1, 3.3]	5.2 [1.9, 13.1]	4.1 [1.7, 9.4]	0.023
**Took medicines**	64.8 [54.2, 74.1]	67.7 [48.3, 82.4]	72.3 [57.4, 83.4]	60.0 [44.6, 73.6]	0.315
**Took antibiotics**	17.1 [13.6, 21.3]	19.8 [11.7, 31.5]	15.5 [9.2, 25.0]	16.9 [11.8, 23.6]	0.705

* Pearson chi-square

** Traditional healer, ordinary shop, household member, friend, neighbor.

When asked about severity of the acute illness, nearly two-thirds of the respondents reported it was a *somewhat* serious condition, while only 9% perceived the condition as *very* serious (severe) and one-quarter as *not* serious. About 7 in 10 (69%) of those with acute illness sought health care outside their homes, a third visited a public facility, and a third visited ADDOs. In 17% of the cases, the ADDO was the first source of care, unless it was a child under five years. However, of the 581 medicines that people received for their acute illnesses, 49% came from an ADDO, 27% from a public facility, 18% from a private facility or pharmacy, and 5% from somewhere else, such as home, neighbors, or family (Table 2).

**Table 2:** Sources of Current Chronic Illness Medicines.

Prescribed/recommended by:	All medicines	Antimicrobials	p-value*
	n = 354 medicines for 287 individuals	n = 36 medicines for 27 individuals	
	Weighted % [95%CI]	Weighted % [95%CI]	
Doctor/nurse	91.7 [87.3, 94.6]	95.6 [73.3, 99.4]	0.427
ADDO dispenser	4.8 [2.4, 9.3]	3.5 [0.3, 28.7]	0.765
Self/household member/friend	1.4 [0.4, 4.4]	0.0 [—, —]	0.522
Other	2.1 [0.6, 7.2]	0.9 [0.1, 9.0]	0.415

* Pearson Chi-square testing difference between antimicrobials and non-antimicrobials

#### Chronic illness

When interviewing the household respondent, the data collector described chronic illness as “an illness that will not go away or takes a long time to go away, even when treated.” Respondents in 252 (21%) of the households reported at least one member with a chronic illness, totaling 288 chronically ill individuals with 344 conditions (Table 3). Among those with a chronic condition, ulcer and chronic stomach pain (15%), arthritis and chronic body pains (15%), followed by high blood pressure (12%) were the most common, particularly among those over 50 years (p≤0.01). Among those in the youngest age group (<25 years) with a chronic condition, asthma/difficulty in breathing (21%) was the most frequently reported.

**Table 3:** Characteristics of Household Members with Chronic Conditions by Age.

Individuals with chronic condition(s)	All	<25 years	25–50 years	>50 years	p-value*
	n = 288	n = 68	n = 111	n = 109	
	Weighted % [95%CI]	Weighted % [95%CI]	Weighted % [95%CI]	Weighted % [95%CI]	
**Region**					
Mbeya	54.8 [31.2, 76.5]	50.1 [23.5, 76.7]	47.0 [25.5, 69.7]	63.1 [36.0, 83.8]	0.125
Morogoro	18.0 [9.3, 31.8]	20.0 [9.0, 38.6]	19.7 [10.6, 33.7]	15.6 [6.1, 34.4]	0.648
Singida	8.0 [3.6, 16.9	7.3 [3.3, 15.1]	12.0 [5.0, 26.0]	5.4 [2.0, 13.8]	0.056
Tanga	19.2 [10.5, 32.5]	22.6 [10.8, 41.4]	21.3 [11.5, 36.0]	15.9 [7.3, 31.4]	0.405
**Chronic condition**					
Arthritis, chronic body pain	15.1 [7.6, 27.7]	4.4 [1.0, 16.8]	10.0 [3.6, 24.6]	24.2 [11.4, 44.0]	0.004
Ulcer, chronic stomach pain	15.2 [7.5, 28.4]	4.3 [0.9, 18.6]	31.7 [18.6, 48.6]	7.9 [1.7, 30.6]	0.004
Hypertension, high blood pressure	12.3 [4.8, 28.1]	0.4 [0.0, 4.8]	10.5 [4.7, 22.0]	19.3 [5.7, 48.7]	0.007
Asthma, wheezing, chronic difficulty breathing	11.9 [5.8, 22.8]	20.5 [8.9, 40.6]	11.0 [5.4, 21.2]	8.3 [2.3, 26.7]	0.146
HIV infection, AIDS	8.5 [4.6, 15.0]	3.8 [1.0, 13.7]	11.1 [5.6, 20.0]	8.8 [2.7, 25.3]	0.421
Diabetes, high blood sugar	6.9 [3.6, 12.7]	0.6 [0.1, 6.6]	2.9 [0.6, 12.6]	13.0 [6.6, 23.9]	0.007
Epilepsy, seizures, fits	12.1 [5.0, 26.2]	13.3 [2.7, 46.0]	12.9 [2.7, 44.2]	10.8 [2.3, 39.0]	0.911
Heart disease, heart attack consequence	4.7 [1.6, 13.2]	10.1 [1.3, 48.5]	4.4 [1.5, 12.4]	2.3 [0.6, 8.6]	0.263
**Usually takes medicines for this condition**	92.8 [84.9, 96.7]	92.8 [80.7, 97.6]	90.3 [77.2, 96.3]	94.6 [85.2, 98.1]	0.447
**Economic reasons for not taking medicines**	2.0 [0.7, 5.9]	3.2 [0.4, 21.4]	1.4 [0.2, 9.2]	1.9 [0.5, 7.7]	0.754

* Pearson chi-square

Almost all of those with chronic illness were reported to be taking medicines for the condition at the time of the survey (93%). Prescribers for chronic disease medicines were usually doctors or nurses (92%) (Table 3). Over 10% of the chronic illness cases had been prescribed antimicrobials, which was more than any other single class of medicine, and those prescriptions came mostly from medical personnel (96%). Ninety-three percent of chronically ill patients reported taking their medicines as recommended. For the few who did not, the most common reasons reported were “symptoms got better” (40%), “no one in the household can take time to obtain medicines” (32%), “cannot afford the medicines” (28%), and “medicine not available in ADDO” (23%).

### Community source of medicines

Data collectors asked to see the drugs that people had on hand at home. Out of the 1,185 households, 422 (36%) had medicines to show the data collector, for a total of 771 samples (Table 4 and S3 Table). Most medicines kept at home were antimicrobials (34%) or analgesics/antipyretics (24%), with the rest divided primarily among antimalarials, anti-cough medications, and antihistamines. Thirty-five percent of the medicines were for current use, while respondents reported that 61% of the medicines had been left over from previous treatment or were being stored for future treatment, including 69% of the antimicrobials and 54% of the antimalarials. A doctor or nurse had recommended 48% of these antimicrobials, while ADDO dispensers had recommended 35%. However, 59% of the antimicrobials had been purchased in ADDOs, 24% came from public facilities, 14% came from private facilities, and 3% from other sources.

**Table 4:** Sources of Medicines from Households.

	Medicines for recent acute illness			Medicines found at home		
	All	Antimicrobials	p-value*	All	Antimicrobials	p-value*
	n = 581	n = 128		n = 771	n = 214	
	Weighted % [95%CI]	Weighted % [95%CI]		Weighted % [95%CI]	Weighted % [95%CI]	
**Prescribed/recommended by:**						
Doctor/nurse	58.1 [47.5, 68.0]	54.7 [36.5, 71.7]	0.683	48.8 [34.8, 62.9]	47.9 [32.9, 63.3]	0.675
ADDO dispenser	33.0 [23.5, 44.1]	38.7 [23.4, 56.7]	0.915	28.5 [17.1, 43.5]	34.9 [23.1, 48.9]	0.011
Other	8.9 [5.9, 13.3]	6.6 [2.1, 18.8]	0.362	22.8 [18.2, 28.1]	17.2 [10.6, 26.7]	0.079
**Obtained at:**						
ADDO	48.7 [35.7, 61.9]	46.8 [23.4, 71.8]	0.809	55.6 [43.5, 67.1]	59.1 [42.5, 73.9]	0.259
Public health facility	29.1 [16.6, 46.0]	38.6 [15.0, 69.2]	0.204	23.4 [16.3, 32.5]	23.6 [11.2, 42.9]	0.995
Private facility (profit or nonprofit)	17.5 [9.7, 29.4]	13.2 [4.7, 32.0]	0.384	17.8 [13.3, 23.4]	14.3 [7.6, 25.2]	0.312
Elsewhere	4.7 [1.7, 12.3]	1.4 [0.2, 8.2]	0.103	3.2 [1.0, 9.7]	3.0 [0.4, 18.4]	0.887

* Pearson chi-square testing difference between antimicrobials and non-antimicrobials

Respondents were also asked about who recommended the medicines used to treat the recent illness. Over half (58%) of the medicines that a doctor or a nurse had recommended were obtained from public health facilities, followed by ADDOs (33%). All of the medicines recommended by ADDO dispensers were bought at an ADDO.

The household survey data indicate that people buy most of their medicines from ADDOs no matter who recommended the treatment. For example, based on ADDO dispensing records, 63% of antimicrobials are dispensed on a prescription; however, if household survey respondents reported receiving a recommendation from someone other than a health care provider or ADDO dispenser (e.g., family member, friend), then over two-thirds of the time (68%), they purchased it at the ADDO. Generally, 62% of household respondents felt that ADDO dispensers give correct advice about treatment; in addition, about 66% felt that ADDOs are the most convenient place to seek care in the community, and 56% said that the ADDO closest to their household usually have the medicines they need. On the other hand, only 37% said that the public health facility has the medicines needed, while 47% thought the quality of care there was good.

### Medicine availability

The health facility and ADDO audits assessed the availability of a list of tracer medicines on the day of the visit. In a comparison of popular antimicrobials, while the availability of capsules and tablets in ADDOs and health facilities was similar, ADDOs had significantly better availability of suspensions and syrups, which are commonly prescribed to treat young children (70% vs. 16%, p<0.001) (Table 5). However, an average of 91% of ADDOs had one or more antimalarials in stock compared with 97% of health facilities (p = 0.072).

**Table 5:** Percent Availability and Median Price Ratios of Tracer Medicines by Facility Type.

	% Availability [95% CI]			Median MPR (25th, 75th percentile)*	
	ADDOs (n = 94)	Health facilities (n = 72)	p-value	ADDOs (n = 94)	Health facilities (n = 72)
**Antimicrobials (Adult formulations)**					
Amoxicillin trihydrate 250 mg, caps	86.8 [74.5, 93.7]	90.9 [79.9, 96.1]	0.431	2.25 [1.69, 2.81)	1.69 [1.35, 3.37)
Co-trimoxazole 480 mg, tablets	91.4 [79.2, 96.7]	79.8 [59.1, 91.5]	0.145	2.65 [2.12, 2.65)	1.75 [1.59, 5.31)
Metronidazole 200mg, tablets	94.6 [88.2, 97.6]	93.4 [82.6, 97.7]	0.750	3.28 [2.95, 4.92)	3.25 [1.64, 4.92)
Erythromycin 250 mg, tablets	83.2 [69.0, 91.7]	86.5 [71.4, 94.3]	0.630	1.59 [1.59, 1.59)	1.59 [1.06, 1.59)
Doxycycline 100mg, caps/tablets	69.2 [57.1, 79.1]	81.4 [53.1, 94.4]	0.301	4.17 [4.17, 4.17)	4.17 [2.21, 4.17)
Ampicillin 250 mg, caps	23.5 [13.2, 38.3]	9.0 [3.4, 21.7]	0.056	1.93 [1.28, 2.15)	1.60 [1.60, 3.08]
Ciprofloxacin 500mg tablets	28.7 [18.5, 41.6]	87.5 [73.6, 94.7]	<0.001	2.38 [1.59, 3.17)	2.38 [1.59, 3.17)
Ampicillin/ cloxacillin 500mg caps	24.3 [12.6, 41.9]	30.5 [15.7,50.8]	0.607	NA	NA
Tetracycline 250 mg caps	26.8 [17.1, 39.6]	9.3 [3.3, 23.5]	0.028	2.26 [2.26, 2.26)	2.26 [2.26, 2.26)
Procaine penicillin fortified 4MU, powder for injection	63.6 [46.9, 77.5]	69.9 [53.0, 82.6]	0.481	2.23 [2.23, 2.23)	2.23 [1.11, 2.23)
Benzyl penicillin 5MU, powder for injection	56.7 [39.9, 72.0]	89.1 [68.9, 96.8]	0.013	2.44 [2.44, 2.44)	2.44 [1.22, 2.44)
**Antimicrobials (Pediatric formulations)**					
Co-trimoxazole 240mg/5mL, suspension	91.0 [80.6, 96.1]	67.9 [49.7, 81.9]	0.012	1.80 [1.80, 2.40)	1.80 [1.20, 1.80)
Erythromycin 125mg/5mL, suspension	79.1 [66.4, 88.0]	57.4 [40.6, 72.6]	0.020	1.33 [1.33, 1.67)	1.00 [1.40, 2.00)
Amoxicillin trihydrate 125mg/5mL, suspension	92.2 [80.2, 97.2]	75.9 [56.0, 88.6]	0.006	2.20 [1.84, 2.45)	1.22 [0.73, 1.96)
Metronidazole 200mg/5mL, suspension	86.2 [75.0, 92.9]	28.1 [15.8, 45.0]	<0.001	3.24 [2.43, 3.24)	2.43 [2.43, 3.24)
**All antimicrobials in stock**	4.3 [1.0, 16.1]	0.0 [., .]	0.239		
**All pediatric formulations in stock**	70.3 [55.4, 81.9]	16.8 [7.3, 34.2]	<0.001		

* Median price ratio (MPR) = median unit price/ MSH unit supply price.

In interviews with 1,365 patients or caretakers exiting outpatient clinics in the four regions, patients had been prescribed 3,413 different types of medicines with an average of 2.5 drugs per prescription. However, the facilities actually dispensed only 2,287 of the prescribed medicines (67%), ranging from only 20% of the prescribed medications dispensed in Morogoro up to 95% of medications dispensed in Singida. For those leaving the clinics with unfilled prescriptions, 45% reported that they planned to buy the undispensed medicines somewhere else, although with substantial variation among regions—Tanga (76%), Mbeya (34%), Morogoro (62%), and Singida (9%).

### Medicine costs and affordability

For the tracer list of medicines (minus antimalarials, which are subsidized at ADDOs), data collectors recorded prices charged per unit at ADDOs and health facilities (e.g., tablet, bottle, ampoule). Public health facilities are directed to sell medicines at half the cost charged by the Medical Stores Department, the parastatal drug supplier. However, the average of the median price ratios for 14 medicines was 2.13 in public facilities rather than something near 0.5, which would be expected if they charged 50% of the supplier price, with a range from 1.00 for erythromycin 250mg (median price across facilities equal to the international reference price) to 4.17 for doxycycline 100mg (317% more than the supplier price) (Table 5).

The average prices paid by patients for the tracer items were generally slightly higher at ADDOs than at health facilities; the ADDO median price ratio of 2.41 was about 13% higher (p = 0.015). Differences existed across regions, but varied by product. For example, in Mbeya antimicrobials in ADDOs were 30% more expensive than in public health facilities, while they were 4% more expensive in public health facilities than ADDOs in Tanga; suspensions in Mbeya were 1% less costly in ADDOs, but 60% more expensive in ADDOs in Morogoro [18]. Using the regimen recommended in Tanzania’s standard treatment guidelines as an illustration [19], the average cost to treat pneumonia in an adult would be TSh444 based on prices of medicines at a health facility (~US$0.26) and TSh528 at an ADDO (~US$0.30) (amoxicillin); and treating a sexually transmitted infection would cost TSh1398 at a health facility (~US$0.81) and TSh1470 at an ADDO (~US$0.85) (benzyl penicillin + co-trimoxazole).

In the household survey, 70% of those interviewed knew that identical medicines could be sold at different prices, but only 51% knew where to find the cheapest prices for medicines or said that it was easy to find out how much medicines cost at different outlets (50%). Most (83%) thought that better quality medicines cost more, while 70% of respondents felt that medicines were more expensive at ADDOs than at the public health facility. In terms of affordability, 49% of respondents said that they had had to sell things or borrow money to pay for medicines at some time in the past, while only 55% reported that the household could usually afford to buy needed medicines.

### Dispensing practices

When data collectors examined the medicines kept in homes (most of which had come from an ADDO), over 90% were adequately labeled with name, treatment duration, and dosage, and over 90% had been packaged in appropriate containers; 100% of the antiretrovirals, which had come from a health facility—most likely a specialized clinic—met all the criteria for appropriate packaging and labeling. During exit interviews at health facilities, data collectors also assessed how the medicines had been packaged and labeled for the patient, and what information the patient or caretaker had received regarding the treatment. At the health facility, only 42% of medicines had been adequately labeled with medicine name, treatment duration, and dosage, but 70% of exiting patients or caretakers knew what the dosage was for the treatment they had received and the duration of treatment.

Mystery shoppers also recorded what they were advised during their ADDO visits (Table 6). Those shoppers presenting the pneumonia scenario received more advice about the nature of the child’s illness than those presenting with cold symptoms (61% vs. 15%; p<0.001). Overall, only 44% of the dispensers asked any of the shoppers about danger signs potentially associated with pneumonia; however, most (55%) of the dispensers asked about one or more of the danger signs when the shopper presented the scenario of a child with pneumonia symptoms (p = 0.003) [20]. Only 29% of shoppers received instructions on how to take the medications they were sold; however, a larger percentage of those in the pneumonia group (34%) were given instructions compared with those who requested an antimicrobial (23%; p = <0.001). Only 5% of shoppers overall were given information about side effects.

**Table 6:** Practices of ADDO dispensers by mystery shopper scenario.

ADDO dispenser practices	Pneumonia (n = 102)	Mild ARI (n = 102)	Septrin request (n = 102)	p-value*
**Probed for danger signs**	55%	45%	31%	0.003
**Asked about:**				
Duration of illness	47%	36%	14%	<0.001
Previous medicines for illness	36%	29%	11%	<0.001
History of similar illness	18%	14%	2%	0.001
Previous visit to health worker for illness	16%	5%	1%	<0.001
Child's weight	14%	9%	5%	0.091
Affordability of recommended medicine	13%	17%	3%	0.005
**Gave a diagnosis**	61%	22%	16%	<0.001
**Dispensed antimicrobials**	54%	34%	85%	<0.001
**Dispensed medicines**	72%	93%	93%	<0.001
**Gave reason for dispensing**	51%	46%	39%	0.262
**Gave instructions on how to use medicine(s)**	34%	29%	23%	<0.001
**Advised caregiver to watch for danger signs**	24%	13%	5%	<0.001

*Chi-square test

### Referrals

ADDO dispensers are trained to refer customers to health facilities in certain circumstances, such as if a child is showing danger signs (as per Integrated Management of Childhood Illness guidelines) or if the health problem is complex. All ADDOs are supposed to receive referral forms free of cost from the regulatory authority; however, of the ADDOs surveyed, only 35% had the forms, and of those, only 16% used them. Over three-quarters of the dispensers interviewed (76%) said that they refer patients with complicated health problems to nearby health facilities: most commonly for severe malaria (81%); severe diarrhea (51%); and high fever (35%). Forty-two percent said that children under five years are the most common category of patients referred.

In the household survey, of the 93 people who went to an ADDO as their first source of care for an acute illness, 28 (30%) were referred to a public facility for a diagnosis. In the mystery shopping exercise, 99% of mystery shoppers presenting a case of pneumonia received an antimicrobial, were referred to a health facility, or both—which are recommended practices for managing pediatric pneumonia.

When interviewers asked health facility prescribers about ADDOs, 88% said they knew of an ADDO in the area, and 72% said they referred patients to ADDOs to buy medicines if the facility was out of stock. However, only 7% were aware of receiving referrals from ADDOs, and only 3% of facilities had a mechanism in place to track referrals.

## Discussion

Our results affirm that ADDOs are the principal source of medicines in Tanzania as well as important suppliers of health care services in the community. This aligns with many studies that have shown the critical role that retail drug sellers play in the health systems of many resource-limited countries [21]. Tanzania’s health system has been designed for people to seek both care and medicines at public health facilities. Our results reflect a situation that is more complicated; for many reasons, people choose not to go to public facilities when they get sick, including feeling the quality of service is poor or not wanting to spend time waiting in line or pay for a consultation, especially when medications are frequently unavailable [22]. In those cases, people may either self-treat with medicine they have stored at home or go to the ADDO for advice or to request a medicine that they had received previously. Although community members and government stakeholders perceived correctly that ADDO drug prices were more expensive; in fact, the differences are fairly small, meaning that ADDOs may cost less after factoring in potential transport and consultation costs and the indirect costs associated with long waiting times. For those who did go to a health facility for an acute illness, most ended up taking their prescriptions to ADDOs to fill, suggesting that the facility was out-of-stock. This aligns with other data on the percentage of ADDO sales that are based on prescriptions [11].

In terms of frequently reported conditions, households in the four study regions reported fever and symptoms of acute respiratory infections as the most common acute illnesses. Although most community members in Tanzania, even in low-transmission areas, still assume that fever is caused by malaria, malaria incidence is declining [23, 24]; the percentage of confirmed malaria cases has come down over 40% since 2005 [25]. Recent research showed that of 1,005 febrile children in Tanzania, only 9% had malaria [26]. A pilot to assess ADDOs as a place for people to receive rapid diagnostic tests for malaria was conducted in two districts where dispensers successfully learned to perform and read the tests and dispense the correct treatment accordingly; in addition, antimicrobial use did not increase, as feared [27]. Uganda experienced similar results when introducing rapid diagnostic tests for malaria in drug shops [28].

Our mystery shopping results that illustrated ADDO dispensers’ ability to handle a child’s case of pneumonia reiterates their ability to provide appropriate care and medicines for a life-threatening illness, although other dispensing practices deviated from their training, particularly related to providing antibiotics when asked for them. Almost two-thirds of dispensers (61%) reported dispensing antibiotics for non-pneumonia coughs because it was just “common practice,” but only 17% said they had been pressured by owners to increase profits by selling more antimicrobials [16]. However, dispensers’ practices are also influenced by patient demand and the quality of prescribing at health facilities. Most of the 56 ADDO dispensers that we interviewed said that customers prefer to purchase antimicrobials for coughs and colds (86%) or for non-bloody diarrhea (73%) [16]. Similarly, most said that they get inappropriate antimicrobial prescriptions for coughs (63%) and for simple diarrhea (45%), but the majority (68%) end up dispensing what is prescribed, even if they know it is wrong.

National and local government officials who were interviewed about services at health facilities and ADDOs recognized the breadth of challenges in improving medicine use in the community. The majority (85%) said the ADDO program has increased access to quality medicines; although 62% thought that the accreditation training improved dispensers’ practices, 74% still thought that there was a lack of compliance with dispensing regulations. On the other hand, 88% thought that prescribers at public health facilities did not adhere to standard treatment guidelines and that health workers’ skills were limited (88%). Almost all (96%) said that poor prescribing and dispensing practices drive antimicrobial resistance, but they also understood the impact from patients who self-medicate (88%) or do not adhere to treatment (71%).

As life expectancies increase and mortality rates from communicable diseases decrease in resource-limited countries, noncommunicable diseases come into sharper focus; in 2013, the United Nations endorsed a global action plan for the prevention and control of noncommunicable diseases [29]. In Tanzania, the proportion of the population over 60 years increased 6% between 2002 and 2012 [30]. In addition, 31% of the population is estimated to have hypertension, and cardiovascular diseases account for 9% of deaths [31]. Although our data showed that almost a quarter of the households included someone with a chronic condition, the ADDO list of allowable prescription medicines has only a handful to treat chronic diseases. For example, of the 18 possible drugs recommended for step-wise treatment of hypertension in Tanzania, ADDOs are only allowed to sell two [19]. As Tanzania develops its approach to detect and manage noncommunicable disease, ADDOs and other private sector providers can provide a valuable contribution [32]. Training ADDO dispensers in the principles of managing important chronic illnesses such as hypertension, diabetes, and asthma, and expanding the ADDO list of chronic disease medicines could potentially contribute to long-term treatment adherence and improved health outcomes.

When the ADDO program was introduced, critics were concerned that allowing drug shops to sell antimicrobials would increase inappropriate medicine use in the community. Previous data have shown that the ADDO program has improved appropriate treatment, although there is still much room for improvement [6, 18]. However, when looking at the entire picture of medicines use in the community, this study supports the view that ADDOs are a key part of an interconnected, multi-faceted system. ADDOs dispense over half of their medicines based on prescriptions from health facilities, and dispensers reported that according to their training, many of these prescriptions are inappropriate. Some dispensers did report sending customers back to the health facility for a new prescription, but more often, they dispensed what was prescribed. Additionally, the fact that many households kept antimicrobials and other medicines on hand for future use suggests a habit of self-medication, which is consistent with other similar evidence in the region [33–35]. WHO includes self-medication as a major factor contributing to irrational use of medicines [36]. Interventions to improve appropriate use of medicines for both acute and chronic illnesses need to integrate the patterns of community care-seeking and the interconnectedness of the health system. Focusing solely on ADDO dispensers would ignore community perceptions and practices, as well as public/NGO sector prescribing quality.

Because so many community members bypass the health facility in favor of seeking care at an ADDO, dispensers need to be able to recognize danger signs and complex illnesses and act as a source of needed referrals. Although the formal (paper-based) referral system in ADDOs is not functioning very well, mystery shopper results showed that ADDO dispensers orally recommended to shoppers presenting the pneumonia scenario to take the child to a health facility. The fact that 99% of pneumonia encounters received an antimicrobial (54%), were referred (90%), or both (45%) is reassuring. Interestingly, a previous intervention that trained ADDO dispensers to detect and refer suspected tuberculosis patients showed that some health care providers were reluctant to recognize ADDO referrals, either by not giving priority to the referred patient as they were supposed to do or by totally ignoring the referral form [37]. The discrepancy between the high percentage of ADDO dispensers who reported referring patients and the low percentage of health facility workers who said they see patients who had been referred may be because some patients do not tell the providers who referred them or never actually go to the health facility after the referral; in addition, some dispensers may have given the interviewers what they thought was a desirable response. Following up on referrals from ADDO dispensers was not within the scope of this research; however, such information would help determine the effectiveness of the current system, such as how many referrals actually go to the health facility and what was the response once they got there. A formal reporting system may not be necessary, but further research would need to determine the most efficient approach to referrals.

The strength of this research was the use of multiple data collection methods to assemble a broad picture of the role of ADDOs in the community health system. However, the study also has limitations. The four regions selected do not represent the situation in all of Tanzania, although they do reflect a range of economic circumstances and bracket the range of experience that regions have with the ADDO program. Because of limitations in record keeping, it was impossible to assess the quality of prescribing in public/NGO health facilities or ADDOs in a meaningful way. However, through the facility audit and exit interviews, we were able to assess important aspects of the pharmaceutical supply situation as well as basic characteristics of medicine packaging, labeling, and communication with patients. Although patients selected for the exit interviews represented a convenience sample at the time of data collection, the practices observed are likely to be representative of the general patient population.

## Conclusions

In the 10 years since its inception in Ruvuma region in 2003, the ADDO initiative has resulted in the creation and institutionalization of new health provider cadre in Tanzania’s health care system. Recognizing that community members choose to seek care in retail drug outlets for a variety of reasons, the government of Tanzania has rightfully targeted quality issues in this setting rather than ignoring them. While most people still go to health facilities, especially for chronic illness care, most buy their medicines for acute illnesses at ADDOs, and ADDO dispensers often refer serious cases to the public/NGO sector. However, poor prescribing in health facilities, poor dispensing at ADDOs, and improper patient demand continue to contribute to inappropriate medicines use; developing policies and interventions that address access to and use of medicines in all three areas has the potential to significantly improve health outcomes in Tanzania.

## Supporting Information

S1 Table List of Prescription Medicines Authorized to Stock and Sell in ADDOs and Tracer Items. (DOCX)

S2 Table Characteristics of Household Members by Age. (DOCX)

S3 Table Sources of Medicines and Care during Acute Illness by Area of ADDO Density. (DOCX)

S1 Dataset (ZIP)
