# Supplementary material for: Understanding the Role of Accredited Drug Dispensing Outlets in Tanzania’s Health System
Source: PLoS One. 2016 Nov 8;11(11):e0164332. doi: 10.1371/journal.pone.0164332 (PMC5100953; doi:10.1371/journal.pone.0164332)
Supplement: S1 Table — (DOCX) [file pone.0164332.s001.docx]

**Supporting Information**

**S1 Table. List of Prescription Medicines Authorized to Stock and Sell in ADDOs and Tracer Items.^a^**

| **TYPE OF MEDICINE** | **STRENGTH** | **TRACER ITEM** |
| --- | --- | --- |
| **Medicines for Asthma** | |  |
| Aminophylline injection (ampoules) | 25 mg/mL in 10 mL |  |
| **Antibiotics** |  |  |
| Amoxicillin trihydrate capsules | 250 mg, 500 mg | x (250 mg) |
| Amoxicillin trihydrate oral suspension | 125 mg/5 mL, 250 mg/mL | x |
| Benzyl penicillin powder for injection | 3 gm (500,000 IU) in vial | x |
| Co-trimoxazole suspension | 240 mg/5 mL in 100 mL bottle | x |
| Co-trimoxazole tablets | 480 mg | x |
| Doxycycline capsules/tablets | 100 mg | x |
| Erythromycin oral suspension | 125 mg/5 mL, 250 mg/5 mL | x |
| Erythromycin tablets | 250 mg, 500 mg | x (250 mg) |
| Metronidazole tablets | 200 mg, 250 mg, 400 mg | x (200mg) |
| Metronidazole suspension | 200 mg/5 mL in 100mL | x |
| Metronidazole injection |  |  |
| Nitrofurantoin tablets | 50 mg, 100 mg |  |
| Oxytetracycline hydrochloride eye ointment | 5% (w/v), 10% (w/v) |  |
| Phenoxymethyl penicillin suspension | 125 mg/5 mL  250 mg/5 mL in 100mL |  |
| Phenoxymethyl penicillin tablets | 250 mg | x |
| Procaine penicillin fortified | 4 g (400,000 IU) – 4 MU | x |
| Silver sulfadiazine cream | 10 mg |  |
| Chloramphenicol eyedrops/ointment |  |  |
| **Anti-Inflammatory/analgesics** | |  |
| Diclofenac sodium tablets | 25 mg, 50 mg |  |
| Indomethacin capsules | 25 mg |  |
| Hydrocortisone ointment/cream | 1%, 0.5% |  |
| Annusol suppositories |  |  |
| **Anesthetics, local** | |  |
| Lignocaine injection | 1% in 10 mL vial, 2% in 30 mL vial |  |
| **Anti-Fungal** | |  |
| Nystatin oral suspension | 100,000 IU/mL in 30 mL bottle |  |
| Nystatin pessaries | 100,000 IU |  |
| Nystatin skin ointment | 100,000 IU/gm |  |
| Nystatin tablets | 500,000 IU |  |

| **TYPE OF MEDICINE** | **STRENGTH** | **TRACER ITEM** |
| --- | --- | --- |
| Ketoconazole tablets |  |  |
| **Antimalarials** | |  |
| Quinine tablets (sulfate or bisulfate) | 300 mg | x |
| Quinine injection (as dihydrochloride) | 300 mg/mL in 2 mL |  |
| Artemether + lumefantrine tablets/ACT | Artemether 20 mg, lumefantrine 120 mg | x (four pack sizes) |
| **Cardiovascular (Anti-arrhythmic drugs)** | |  |
| Propranolol tablets (hydrochloride) | 10 mg, 40 mg, 80 mg |  |
| **Diuretics** | |  |
| Bendrofluazide tablets | 5 mg |  |
| **Oxytocics** | |  |
| Ergometrine injection (maleate) | 0.2 mg/mL in 1 mL ampoule  0.5 mg/mL in 2 mL ampoule |  |
| **Laxative** | |  |
| Bisacodyl tablets | 5 mg |  |
| **Antihistamines** | |  |
| Cetirizine hydrochloride tablets | 10 mg |  |
| Cetirizine hydrochloride oral solution | 5 mg/5 mL |  |
| Antispasmodics | |  |
| Hyoscine butylobromide tablets | 10 mg |  |
| Hyoscine butylobromide injection | 20 mg/mL |  |
| **Oral Contraceptives** | |  |
| Ethinylestradiol + novethisterone | Ethinylestradiol (0.03mg) + Novethisterone (0.3mg) |  |
| Ethinylestradiol + levonorgestrel | Ethinylestradiol (0.03mg)+ Levonorgestrel (0.15mg) |  |
| **Minerals/vitamins** | |  |
| Neurobion forte |  |  |
| Zinc sulfate tablets | 20 mg |  |
| **Anti-emetic** | |  |
| Promethazine hydrochloride injection | 25 mg/mL in 2 mL ampoule |  |
| **Fluids and Electrolytes** | |  |
| Dextrose | 5% |  |
| Normal saline injection | 0.9% |  |
| Water for injection |  |  |
| **Anti-Epileptic** | |  |
| Phenytoin tablets/capsules (sodium salt) | 50 mg, 100 mg |  |

**^a^** mg = milligram; mL = milliliter, gr = gram, IU = international unit
