# Supplementary material for: Understanding the Role of Accredited Drug Dispensing Outlets in Tanzania’s Health System
Source: PLoS One. 2016 Nov 8;11(11):e0164332. doi: 10.1371/journal.pone.0164332 (PMC5100953; doi:10.1371/journal.pone.0164332)
Supplement: S2 Table — (DOCX) [file pone.0164332.s002.docx]

**S2 Table. Characteristics of Household Members by Age.**

|  | **All** | **<5 years** | **5-14 years** | **15-24 years** | **25-50 years** | **>50 years** |  |
| --- | --- | --- | --- | --- | --- | --- | --- |
| **Household members** | n= 6384 | n= 763 | n= 1696 | n= 1307 | n= 1820 | n= 792 |  |
|  | Weighted % [95%CI] | Weighted % [95%CI] | Weighted % [95%CI] | Weighted Percentage [95%CI] | Weighted Percentage [95%CI] | Weighted Percentage [95%CI] | p-value* |
| **From region** |  |  |  |  |  |  |  |
| Mbeya | 34.1 [20.6, 50.8] | 38.3 [23.4, 55.7] | 32.6 [19.8, 48.7] | 35.7 [21.2, 53.5] | 33.3 [19.9, 50.1] | 32.6 [18.2, 51.2] | 0.352 |
| Morogoro | 27.3 [15.2, 44.0] | 23.1 [12.8, 38.0] | 28.1 [16.1, 44.5] | 26.5 [13.3, 45.7] | 28.6 [16.2, 45.3] | 28.0 [14.4, 47.4] | 0.463 |
| Singida | 19.4 [10.8, 32.4] | 20.6 [11.2, 34.8] | 21.6 [12.1, 35.6] | 17.9 [9.8, 30.3] | 18.5 [10.2, 31.3] | 18.5 [9.8, 31.9] | 0.197 |
| Tanga | 19.2 [10.2, 33.0] | 18.0 [9.5, 31.5] | 17.6 [9.4, 30.6] | 19.9 [10.1, 35.5] | 19.6 [10.7, 33.2] | 20.9 [10.5, 37.3] | 0.446 |
| **Male** | 48.0 [46.6, 49.5] | 54.0 [49.8, 58.2] | 51.1 [48.2, 54.0] | 46.4 [43.0, 49.9] | 42.0 [39.8, 44.2] | 52.3 [48.2, 56.4] | <0.001 |
| **Acute illness in past 2 weeks** | 9.8 [8.5, 11.3] | 16.9 [12.4, 22.7] | 9.2 [7.1, 11.9] | 5.7 [4.3, 7.4] | 9.5 [7.8, 11.5] | 11.7 [9.3, 14.6] | <0.001 |
| **Diagnosed chronic condition** | 4.6 [4.0, 5.3] | 0.5 [0.2, 1.2] | 1.7 [1.1, 2.6] | 2.2 [1.4, 3.3] | 6.0 [4.7, 7.5] | 14.6 [10.7, 19.7] | <0.001 |
| **Household has 6+ members** | 52.4 [46.0, 58.7] | 50.9 [42.7, 59.1] | 61.4 [55.3, 67.3] | 57.9 [49.6, 65.8] | 45.2 [39.3, 51.3] | 42.7 [35.6, 50.1] | <0.001 |
| **Enrolled in health insurance** | 18.5 [14.6, 23.2] | 15.8 [11.5, 21.3] | 16.9 [13.0, 21.8] | 21.6 [16.7, 27.5] | 17.6 [13.6, 22.4] | 21.1 [15.7, 27.7] | 0.024 |
| **Where medicines were found** | 37.1 [31.6, 42.8] | 42.1 [33.2, 51.6] | 37.1 [30.4, 44.3] | 36.6 [30.7, 42.8] | 38.2 [32.7, 44.0] | 31.0 [25.7, 36.7] | 0.060 |
| **Where antibiotics were found** | 15.7 [12.3, 19.8] | 20.7 [14.6, 28.6] | 16.0 [11.9, 21.0] | 15.3 [11.4, 20.2] | 15.5 [12.0, 19.7] | 11.7 [8.2, 16.3] | 0.040 |
| * Pearson chi-square |  |  |  |  |  |  |  |
