# Supplementary material for: Understanding the Role of Accredited Drug Dispensing Outlets in Tanzania’s Health System
Source: PLoS One. 2016 Nov 8;11(11):e0164332. doi: 10.1371/journal.pone.0164332 (PMC5100953; doi:10.1371/journal.pone.0164332)
Supplement: S3 Table — (DOCX) [file pone.0164332.s003.docx]

**S3 Table. Sources of Medicines and Care during Acute Illness by Area of ADDO Density.**

|  | **Sources of medicine** | | | | | |
| --- | --- | --- | --- | --- | --- | --- |
|  | **Public facility** | **ADDO** | **Private facility** | **Other** | **None** | **Total** |
| **ADDO density*** | **n (%)** | **n (%)** | **n (%)** | **n (%)** | **n (%)** | **n (%)** |
| **High** | 81 (28.7) | 135 (47.9) | 34 (12.1) | 32 (11.4) | N/A | 282 (100) |
| **Low** | 73 (34.8) | 96 (45.7) | 18 (8.6) | 23 (10.9) | N/A | 210 (100) |
| **None** | 30 (33.3) | 43 (47.8) | 6 (6.7) | 11 (12.2) | N/A | 90 (100) |
| **Total** | 184 (31.6) | 274 (47.1) | 58 (10.0) | 66 (11.3) | N/A | 582 (100) |
| Chi-square = 4.337, p = 0.631 | | | | | | |
|  | **Sources of care** | | | | | |
| **High** | 86 (30.2) | 52 (18.3) | 33 (11.6) | 50 (17.5) | 64 (22.5) | 285 (46.8) |
| **Low** | 74 (32.0) | 38 (16.5) | 24 (10.4) | 38 (16.5) | 57 (24.7) | 231 (37.9) |
| **None** | 32 (34.4) | 10 (10.8) | 8 (8.5) | 17 (18.3) | 26 (27.9) | 93 (15.3) |
| **Total** | 192 (31.5) | 100 (16.4) | 65 (10.7) | 105 (17.2) | 147 (24.1) | 609 (100) |
| Chi-square = 4.5175, p = 0.808 | | | | | | |

*High-density wards (administrative unit smaller than a district) in all regions except Singida had ≥5 ADDOs; Singida region had fewer ADDOs, so high-density wards had ≥3 ADDOs. Low-density wards included all other wards with ADDOs. Some wards had no registered ADDOs, and presumably, people in those wards travel to neighboring wards for access.
